# Characterization of small plasmids carrying florfenicol resistance gene *floR* in *Actinobacillus pleuropneumoniae* and *Pasteurella multocida* isolates from swine in China

**DOI:** 10.3389/fvets.2023.1084491

**Published:** 2023-01-30

**Authors:** Xiaohui Yao, Qiangqiang Song, Wei Zhu, Jianchao Wei, Donghua Shao, Ke Liu, Zongjie Li, Yafeng Qiu, Zhiyong Ma, Lining Xia, Beibei Li

**Affiliations:** ^1^Shanghai Veterinary Research Institute, Chinese Academy of Agricultural Sciences, Shanghai, China; ^2^College of Veterinary Medicine, Xinjiang Agricultural University, Urmuqi, China; ^3^Tengzhou Animal Disease Prevention and Control Center of Shandong Province, Tengzhou, China

**Keywords:** pig, *floR*, PFGE, *Pasteurellaceae*, antibiotic resistance

## Abstract

*Actinobacillus pleuropneumoniae* and *Pasteurella multocida* are two important bacterial pathogens in swine industry. In the present study, resistance profiles of nine commonly used antibiotics of *A*. *pleuropneumoniae* and *P*. *multocida* isolates of swine origin from different regions of China were investigated by determination of minimum inhibitory concentrations (MICs). In addition, genetic relationship of the florfenicol-resistant *A*. *pleuropneumoniae* and *P*. *multocida* isolates was determined by pulsed-field gel electrophoresis (PFGE). The genetic basis of florfenicol resistance in these isolates were explored by *floR* detection and whole genome sequencing. High resistance rates (>25%) of florfenicol, tetracycline and trimethoprim- sulfamethoxazole were observed for both bacteria. No ceftiofur- and tiamulin- resistant isolates were detected. Furthermore, all the 17 florfenicol-resistant isolates (nine for *A*. *pleuropneumoniae* and eight for *P*. *multocida*) were positive for *floR* gene. The presence of similar PFGE types in these isolates suggested that clonal expansion of some *floR*-producing strains occurred in the pig farms from same regions. WGS and PCR screening showed that three plasmids, named pFA11, pMAF5, and pMAF6, were the cargos of the *floR* genes in the 17 isolates. Plasmid pFA11 exhibited novel structure and carried several resistance genes, including *floR, sul2, aacC2d, strA, strB*, and *bla*_ROB − 1_. Plasmids pMAF5 and pMAF6 were presented in *A*. *pleuropneumoniae* and *P*. *multocida* isolates from different regions, suggesting horizontal transfer of the two plasmids are important for the *floR* dissemination in these *Pasteurellaceae* pathogens. Further studies of florfenicol resistance and its transfer vectors in *Pasteurellaceae* bacteria of veterinary origin are warranted.

## Introduction

*Actinobacillus pleuropneumoniae* and *Pasteurella multocida*, two members of the family *Pasteurellaceae*, are important pathogens and cause significant economic losses in swine industry worldwide (1). *A*. *pleuropneumoniae* is etiological agent of porcine contagious pleuropneumonia and could be divided into two biovars based on the NAD dependence (2). To date, a total of 19 serovars has been described, and of them, serovars 1, 5, 9, and 11 are highly virulent and frequently observed in the clinical cases of porcine pleuropneumonia (3). *P*. *multocida* is commensal bacterial inhabitant of the upper respiratory tract of pigs and associated with atrophic rhinitis and pneumonia in clinical diseased pigs (4). Besides pigs, *P*. *multocida* also causes different diseases in various animals, such as cattle, poultry, rabbit and pets, and even humans (5, 6). Using molecular typing methods, *P*. *multocida* isolates could be classified into five capsular serotypes (A, B, D, E, F) and eight LPS genotypes (L1–L8) (7). Of them, serotypes A and D are most commonly isolated in diseased pigs (8). Due to the diverse serotypes and geographical difference in their prevalence, there is presently no vaccine with satisfactory protection to control *A*. *pleuropneumoniae* and *P*. *multocida* infections (2). For this reason, antibiotic use is still the dominant strategy for the treatment and prevention of the infectious diseases caused by these two pathogenic bacteria. However, due to the massive and long-term antibiotic use in pig farm, resistance to commonly used antibiotics in *A*. *pleuropneumoniae* and *P*. *multocida* isolates has emerged and been increasingly reported all over the world (1, 9).

Florfenicol, one member of phenicol family, is exclusively approved for veterinary medicine. With the structural modification, florfenicol do not have the toxic side effect of chloramphenicol and could avoid the acetyltransferase-mediated drug resistance (10). In China, florfenicol was licensed in 1999 for treatment of bacterial infections in cattle, pig, chicken and fish (11). It is one of the most used antibiotics in food-animal industry and about 10,000 tons was used in China in 2013 (12). Florfenicol is a broad-spectrum antimicrobial drug that are widely used to treat respiratory diseases and other pathogenic bacterial infections in pig farm, including *A*. *pleuropneumoniae* and *P*. *multocida* infections. Florfenicol-resistant *A*. *pleuropneumoniae* and *P*. *multocida* isolates have been sporadically reported in different countries and, same to other Gram-negative bacteria, *floR* is the predominant determinant for resistance to florfenicol in those isolates (8, 9, 13, 14). In these isolates, *floR* was mainly carried by plasmids with various sizes or integrative conjugative elements (ICEs) (9, 15–21).

There are few data about the florfenicol resistance and its genetic basis in *A*. *pleuropneumoniae* and *P*. *multocida* isolates from swine in China. In the present study, we collected *A*. *pleuropneumoniae* and *P*. *multocida* isolates from pig farms in different regions of China, and characterized the genetic relationships of the florfenicol-resistant isolates and the structures of relevant *floR*-carrying plasmids.

## Materials and methods

### Bacterial strains and susceptibility testing

A total of 19 *A*. *pleuropneumoniae* isolates and 31 *P*. *multocida* isolates were used in this study. These isolates were collected between September 2018 to December 2020 in routine clinical diagnosis in several pig farms from different provinces of China, including Jiangsu, Xinjiang, Shanghai and Hubei. Minimum inhibitory concentrations (MICs) were determined by broth microdilution method according to recommendation of the Clinical and Laboratory Standards Institute (CLSI) supplement VET08 (22). Nine commonly used antibiotics were tested, including florfenicol, ceftiofur, enrofloxacin, tetracycline, tiamulin, tilmicosin, gentamicin, ampicillin and trimethoprim-sulfamethoxazole. For *A*. *pleuropneumoniae*, clinical breakpoints were interpreted with CLSI-VET08 document for these antibiotics, except trimethoprim-sulfamethoxazole, for which the Epidemiological Cut-off value (ECOFF) value given by European Committee on Antimicrobial Susceptibility Testing (EUCAST) was temporarily used as resistance breakpoint (https://www.eucast.org/). In addition, CLSI has no clinical breakpoints of tiamulin, gentamicin, and trimethoprim-sulfamethoxazole for *P*. *multocida* and the ECOFF values from EUCAST were also used as resistance breakpoints for these antibiotics. Moreover, MIC_50_ and MIC_90_ were exhibited to reflect the MIC distributions. *Escherichia coli* ATCC 25922 and *A*. *pleuropneumoniae* ATCC 27090 were used as quality control strains.

### Pulsed-field gel electrophoresis and *floR* detection

All the florfenicol-resistant isolates were screened for the presence of *floR* gene with specific primer sets (5′-CTGAACACGACGCCCGCTATG-3′ and 5′-CAGGACCGCTCCGCAAACAA-3′) with the amplification condition: 95°C for 5 min; followed by 30 cycles of 95°C for 30 s, 58°C for 30 s, and 72°C for 60 s; and extension at 72°C for 7 min. Sanger sequencing of the amplicons was performed to confirm the PCR results. To investigate the genetic relationship between the *floR*-positive isolates, *Apa*I-PFGE was performed as described previously with modified running parameter: 14°C, 6 V/cm, 2.16 s initial switching time, 54.2 s final switching time and a 18 h of total run (23, 24). The fingerprinting profiles were analyzed with BioNumerics v7.1 (Applied-Maths, Kortrijk, Belgium) to construct a dendrogram based on the Dice coefficient with 1.0% band-position tolerance and 1.5% optimization. Genetical relatedness between the *floR*-positive isolates were determined with the cut-off of 80% homology of the DNA restriction patterns (25, 26).

### Whole genome sequencing and bioinformatic analysis

The genetically unrelated *floR*-positive three *A. pleuropneumoniae* and three *P. multocida* isolates were further subjected to WGS with the Illumina Hiseq 2000 platform. The raw data was assembled using the SPAdesv.3.13.0. Resfinder was used to analyze resistance genes and to find the *floR*-bearing contigs. Invert PCRs with the primers located at the both ends of the *floR*-bearing contigs were performed and the generated amplicons were subjected to Sanger sequencing, by which the complete sequences of the *floR*-carrying plasmids were obtained. Annotation was automatically generated using the RAST (https://rast.nmpdr.org/) (27). Structure comparisons with the plasmids deposited in Genbank were performed by BLAST (www.ncbi.nlm.nih.gov/BLAST) (28). Moreover, three specific primer sets were designated for the obtained three types of *floR*-carrying plasmids (P1: 5′-AGGTCGCCCTAAACTTCC-3′ and P2: 5′-TATCGCCTGCCATCCC-3′ for pFA11, P3: 5′-AATGGTTACAGGTGGAAGA-3′ and P4: 5′-GCACTGCTGCTGATGG-3′ for pMAF5, and P5: 5′-AGGGCGATTTATGATTGA-3′ and P6: 5′-TCGGCGAACTTTACGG-3′ for pMAF6). PCRs with the three primer sets were conducted to detect the plasmid types in the *floR*-producing isolates without WGS analysis. The amplicons were sequenced and compared with corresponding plasmids to confirm the PCR results.

### Nucleotide sequence accession number

The complete sequences of the *floR*-carrying plasmids pFA11, pMAF5 and pMAF6 have been deposited in the GenBank database with the accession numbers CP100665, CP100664, CP100663.

## Results and discussion

Resistance profiles of the 19 *A. pleuropneumoniae* and 31 *P. multocida* isolates were exhibited in Tables 1, 2. In *A. pleuropneumoniae* isolates, high resistance rates were observed for tetracycline and trimethoprim-sulfamethoxazole (89.5 and 68.4%, respectively), while florfenicol showed moderate degree of resistance (47.4%). Low frequencies of resistance were observed for ampicillin, enrofloxacin, tilmicosin and gentamicin (10.5, 10.5, 5.3, and 5.3%, respectively). No resistance of ceftiofur and tiamulin were detected for these *A. pleuropneumoniae* isolates. In *P. multocida* isolates, highest resistance rate was observed for trimethoprim-sulfamethoxazole (87.1%). Eight and twelve isolates showed resistance to florfenicol and tetracycline, corresponding to resistance rates 25.8 and 38.7%, respectively. For other test antibiotics, no resistance was detected.

**Table 1:** MIC distribution and resistance profiles of nine antibiotics for 19 *A. pleuropneumoniae* isolates.

**Antibiotic**	**MIC(**μ**g/mL)**															**MIC_50_**	**MIC_90_**	**Resistance %**

	<**0.063**	**0.063**	**0.125**	**0.25**	**0.5**	**1**	**2**	**4**	**8**	**16**	**32**	**64**	**128**	**256**	>**256**			
Florfenicol	-	0	0	1	2	7	0	0	3	2	4	-	-	-	-	1	32	47.4%
Ceftiofur	19	0	0	0	0	0	0	0	0	0	0	-	-	-	-	<0.063	<0.063	0.0%
Enrofloxacin	16	0	0	0	1	2	0	0	0	0	0	-	-	-	-	<0.063	1	10.5%
Tetracycline	-	-	0	0	1	1	0	14	2	1	0	-	-	-	-	4	8	89.5%
Tiamulin	-	-	-	-	-	0	0	0	8	11	0	0	0	-	-	16	16	0.0%
Tilmicosin	-	-	-	-	-	0	0	10	7	1	0	0	1	-	-	4	8	5.3%
Gentamicin	-	-	-	-	0	0	6	12	0	0	0	0	0	0	1	4	4	5.3%
Ampicillin	2	0	0	0	15	0	0	0	0	0	2	0	-	-	-	0.5	32	10.5%
Trimethoprim-sulfamethoxazole	1	4	1	5	4	1	0	0	0	0	3	-	-	-	-	0.25	32	68.4%

Thin vertical lines indicate the breakpoints between susceptible and intermediate values. Thick vertical lines indicate the breakpoints between intermediate and resistant values. For tiamulin, tilmicosin and trimethoprim-sulfamethoxazole, thick vertical lines indicate the breakpoints between susceptible and resistant values. White areas indicate range of tested dilutions for each antibiotic; The MIC_50_ and MIC_90_ values are concentrations, at which ≥50% and ≥90% of isolates are inhibited, respectively.

**Table 2:** MIC distribution and resistance profiles of nine antibiotics for 31 *P*. *multocida* isolates.

**Antibiotic**	**MIC(**μ**g/mL)**															**MIC_50_**	**MIC_90_**	**Resistance %**

	<**0.063**	**0.063**	**0.125**	**0.25**	**0.5**	**1**	**2**	**4**	**8**	**16**	**32**	**64**	**128**	**256**	>**256**			
Florfenicol	-	0	0	5	17	0	0	1	0	4	4	-	-	-	-	0.5	32	25.8%
Ceftiofur	31	0	0	0	0	0	0	0	0	0	0	-	-	-	-	<0.063	<0.063	0.0%
Enrofloxacin	21	0	8	2	0	0	0	0	0	0	0	-	-	-	-	<0.063	0.125	0.0%
Tetracycline	-	-	0	0	14	5	8	3	0	1	0	-	-	-	-	1	4	38.7%
Tiamulin	-	-	-	-	-	0	0	2	2	17	10	0	0	-	-	16	32	0.0%
Tilmicosin	-	-	-	-	5	0	11	12	2	1	0	0	0	-	-	2	4	0.0%
Gentamicin	-	-	-	-	0	5	7	19	0	0	0	0	0	0	-	4	4	0.0%
Ampicillin	-	5	0	17	9	0	0	0	0	0	0	0	-	-	-	0.25	0.5	0.0%
Trimethoprim- sulfamethoxazole	-	0	4	1	6	0	1	3	1	12	3	-	-	-	-	8	16	87.1%

Thin vertical lines indicate the breakpoints between susceptible and intermediate values. Thick vertical lines indicate the breakpoints between intermediate and resistant values. For tiamulin, tilmicosin, gentamicin, and trimethoprim-sulfamethoxazole, thick vertical lines indicate the breakpoints between susceptible and resistant values. White areas indicate range of tested dilutions for each antibiotic; The MIC_50_ and MIC_90_ values are concentrations at which ≥50 and ≥90% of isolates are inhibited.

**Figure 1:**
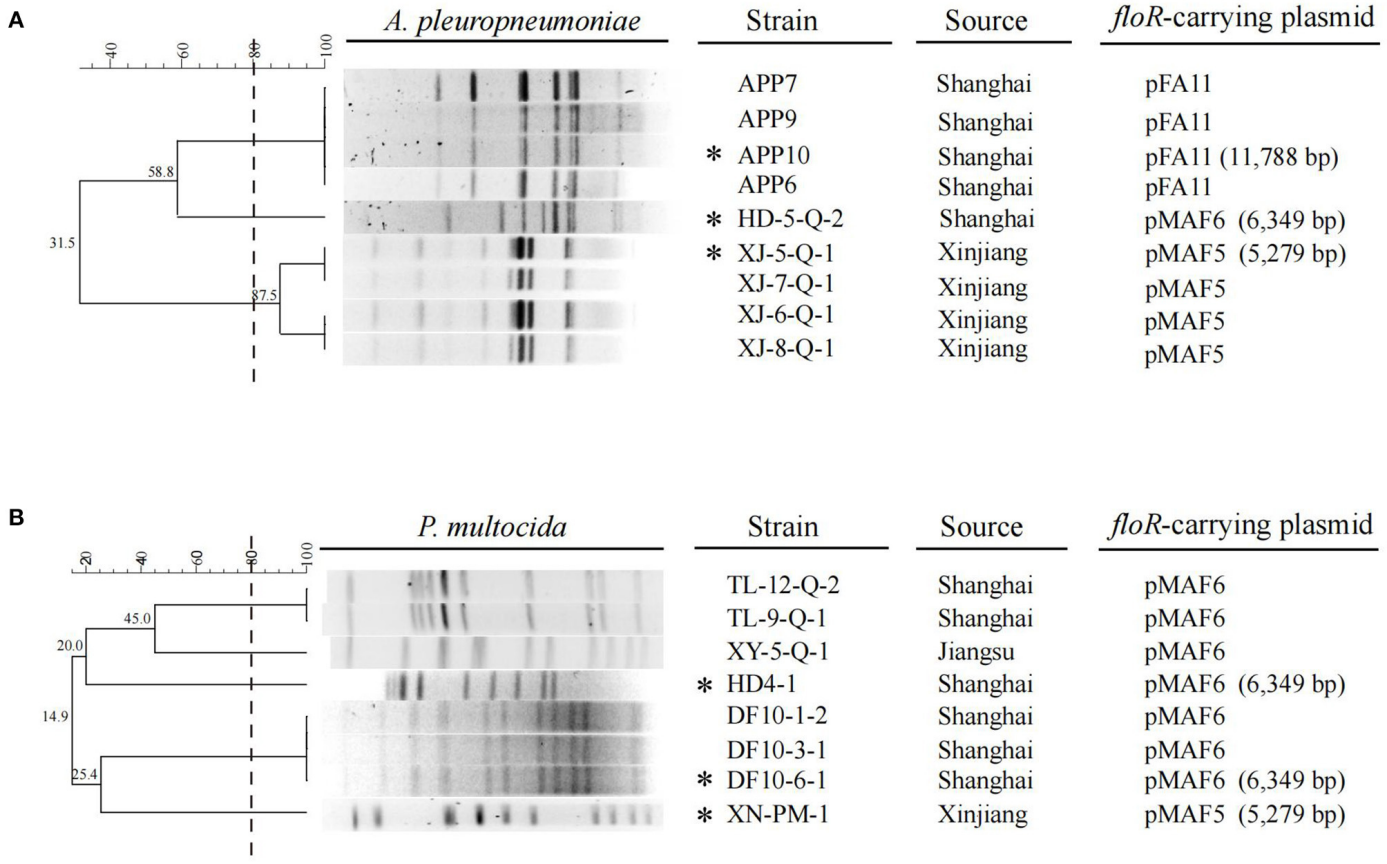
*Apa*I-PFGE patterns and dendrogram of the 9 *floR*-positive *A*. *pleuropneumoniae* isolates **(A)** and 8 *floR*-positive *P*. *multocida* isolates **(B)** in this study. The dashed line on the dendrogram indicates 80% similarity. The strain name, source and the *floR*-carrying plasmid corresponding to each isolate are also presented. The isolates marked with an asterisk are the ones characterized by WGS, while the *floR*-carrying plasmids in the isolates without asterisk are identified by specific PCRs.

Florfenicol is an important antibiotic and extensively used in swine industry. Unlike other Gram-negative bacteria, such as *Enterobacteriaceae*, resistance to florfenicol in *A. pleuropneumoniae* and swine-origin *P. multocida* isolates has rarely been reported all over the world (1, 4, 29–31), except in Korea, where about 36% of *A. pleuropneumoniae* isolates collected from 2006 to 2013, and 16.3% swine-origin *P. multocida* isolates collected between 2008 and 2016 were resistant to florfenicol (13). In China, there is no epidemiological data about florfenicol resistance available for *A. pleuropneumoniae*. In 2009, a study concerning the antimicrobial resistance and virulence genes of swine-origin *P. multocida* isolates in China showed that no florfenicol-resistant strains were detected (4). In the present study, relatively high degree of florfenicol resistance were observed for *A. pleuropneumoniae* and swine-origin *P. multocida* isolates (9/19, 47.4% and 8/31, 25.8%, respectively). All of these strains were positive for *floR* gene. These data indicated that *floR*-mediated florfenicol resistance in *P. multocida* and *A. pleuropneumoniae* isolates have emerged and spread in pig farms of China in recent years. According to our survey, florfenicol is one of the most frequently applied antibiotics in the pig farms where the *P. multocida* and *A. pleuropneumoniae* strains used in this study were recovered. The high selection pressure due to extensive use of florfenicol in these farms may promote the dissemination of *floR* gene and associated resistant isolates. The genetic relationship of these *floR*-producing isolates was investigated by PFGE (Figure 1). The nine *A. pleuropneumoniae* isolates could be divided into three pulsotypes. Four strains from Shanghai showed highly similar band patterns. While another strain HD-5-Q-2 from Shanghai was located on a separated branch. The four isolates from Xinjiang exhibited ≥80% phylogenetic similarity. The eight *P. multocida* isolates showed highly various PFGE patterns. Six isolates from Shanghai exhibited three different types, while the isolates XY-5-Q-1 from Jiangsu and XN-PM-1 from Xinjiang were located on different branches. The same PFGE patterns observed in some isolates from same regions of China suggested that clonal expansion of *floR*-positive *A. pleuropneumoniae* and *P. multocida* isolates exists in these pig farms.

To further investigate the genetic basis of the *floR* transmission in *A. pleuropneumoniae* and swine-origin *P. multocida* isolates, three *A. pleuropneumoniae* and three *P. multocida* strains with different PFGE patterns were subjected to WGS and the complete *floR*-carrying plasmids in these isolates were obtained by PCR-based gap filling. Three types of *floR*-carrying plasmid, named pFA11, pMAF5, and pMAF6, were identified in the six isolates. Furthermore, specific PCRs corresponding to the three plasmids were performed to detect whether these plasmids were also present in other 11 *floR*-producing isolates. Taken together, plasmid pFA11 was detected in four *A. pleuropneumoniae* isolates, pMAF5 served as the carriers of *floR* gene in four *A. pleuropneumoniae* and one *P. multocida* isolates, and pMAF6 was present in one *A. pleuropneumoniae* and seven *P. multocida* isolates (Figure 1). These data demonstrated that the three plasmids mediated *floR* transmission in *A. pleuropneumoniae* and *P. multocida* isolates from swine in China.

**Figure 2:**
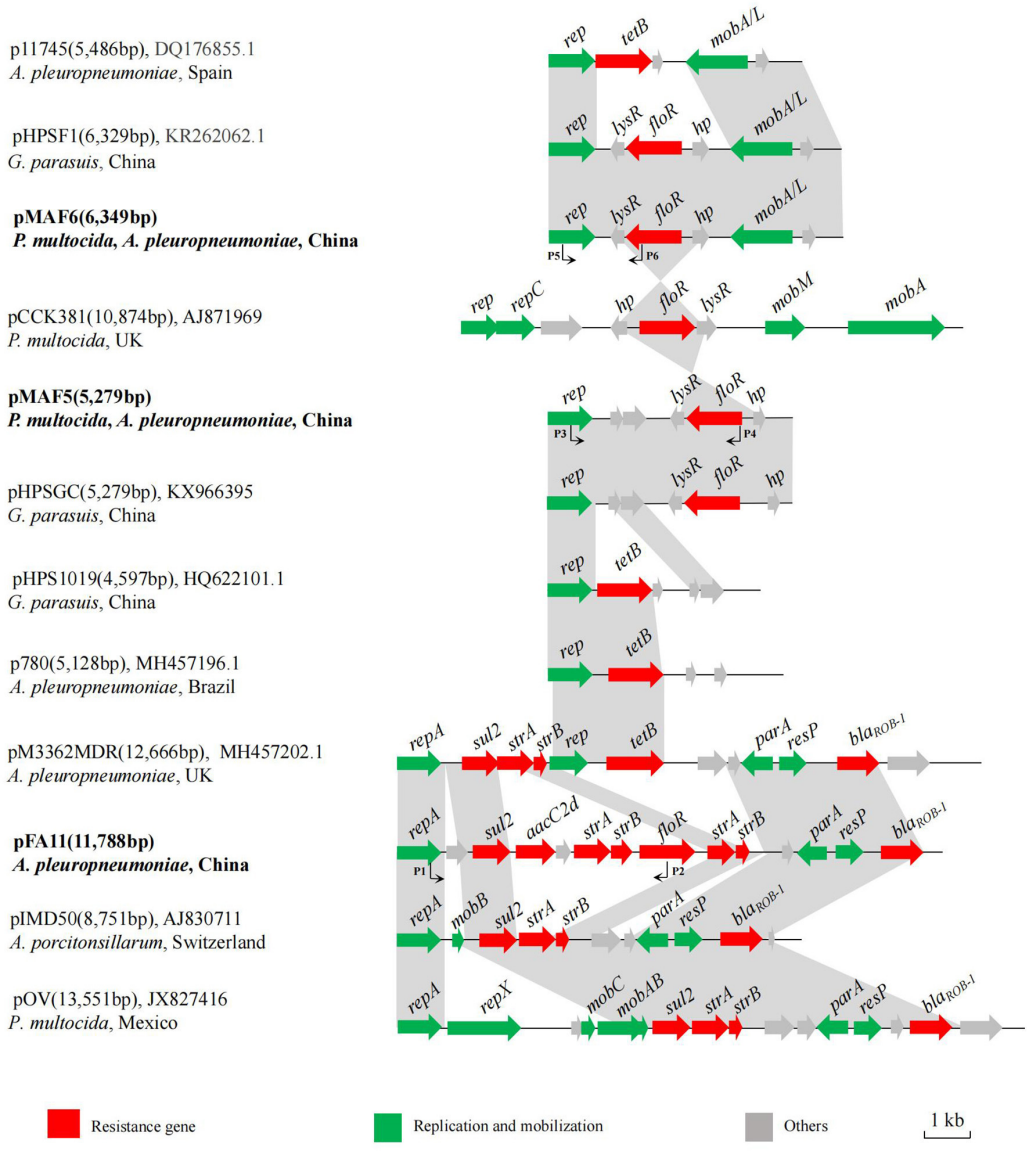
Schematic comparison of the *floR*-carrying plasmids pFA11, pMAF5, and pMAF6 with related plasmids in Genbank. Arrows represented the positions, orientations and relative sizes of the genes. The regions with ≥98% homology between these plasmids are marked by gray shading. The plasmid name, size, GenBank accession number, host bacteria and country of origin are given on the left of each plasmid map.

Plasmid pFA11, identified in four *A. pleuropneumoniae* isolates from Shanghai, was 11,788 bp in size and consisted of 14 opening reading frames (ORFs). Besides *floR*, pFA11 also contained sulfonamide resistance gene *sul2*, aminoglycoside resistance genes *aacC2d, strA, strB*, and β-lactam resistance gene *bla*_ROB − 1_. Comparison with sequences deposited in Genbank showed that pFA11 shared highly structural similarity with plasmids originated from other *Pasteurellaceae* bacteria such as pIMD50 from *A. porcitonsillarum* of swine origin in Switzerland (GenBank accession no. AJ830711) and pOV described in *P. multocida* isolated from Mexico (GenBank accession no. JX827416) (Figure 2) (32, 33). There are two main differences between pFA11 and the latter two plasmids. Firstly, compared to pFA11, additional genes associated with plasmid replication and mobilization were present within the other three plasmids, such as *repA, repX, mobA, mobB* and *mobC*. Secondly, plasmids pIMD50 and pOV shared highly similar resistance gene cluster. While, pFA11 harbored four additional resistance genes, including *floR, aacC2d*, and another copy of *strA* and *strB*. Since the antibiotics corresponding to these resistance genes are commonly used in pig farms, the multiple resistance gene cluster on pFA11 may facilitate the spread of this plasmid in the *A. pleuropneumoniae* isolates through the co-selection effect.

pMAF5 was 5,279 bp in length with simple structure, in which a plasmid replication gene *rep*, a resistance gene *floR* and four putative ORFs were present. Database searching showed that pMAF5 was highly similar to plasmid pHPSGC from *G. parasuis* HpsGC of sick pig in China (GenBank accession no. KX966395) (Figure 2). Structure comparison revealed that *floR* and its flanking region (*lysR*-*floR*-*hp*) showed high homology to the corresponding region of plasmid pCCK381 described in *P. multocida* isolate strain 381 from lung of a calf origin from UK (GenBank accession no. AJ871969) (18). The *rep* gene of pMAF5 was same to those of other resistance plasmids from different members of family *Pasteurellaceae*, such as pHPS1019 from *G. parasuis* lung6591 isolates in China (GenBank accession no. HQ622101), p780 originated from *A. pleuropneumoniae* MV780 in Brazil (GenBank accession no. MH457196) and pM3362MDR from *A. pleuropneumoniae* MID3362 originated from pig lung in UK (GenBank accession no. MH457202) (34). These plasmids possessed other resistance genes, such as *tet* (B), *bla*_ROB − 1_, *sul2* and *strA*, but not *floR*.

Plasmid pMAF6 was the most frequently observed *floR*-vector, which was detected in eight isolates from different regions and with various PFGE patterns. pMAF6 was 6, 349 bp in size and showed >99% nucleotide identity to previously reported *floR*-bearing plasmid pHPSF1 identified from *G. parasuis* ASB17W in China (GenBank accession no. KR262062) (35) (Figure 2). Compared to pHPSF1, pMAF6 had a 22 bp insertion at the end of the putative *lysR* gene, which generated an early stop codon and encoded a smaller putative LysR (101 amino acids for pMAF6 and 109 amino acids for pHPSF1). The same plasmid structure was also observed for plasmid p11745 in *A. pleuropneumoniae* APP11745 from Spain (GenBank accession no. DQ176855), which served as the cargo of tetracycline resistance gene *tet* (B) (36). Moreover, similar to plasmid pMAF5, the fragment *lysR*-*floR*-*hp* of pMAF6 was also highly similar to the corresponding region of plasmid pCCK381.

## Conclusions

The genetic basis of the florfenicol resistance in *A*. *pleuropneumoniae* and *P*. *multocida* isolates of swine origin in China was analyzed in this study. Considering the importance of florfenicol in pig industry, the high prevalence of florfenicol resistance and *floR* gene observed in this study may significantly affect the control of *A. pleuropneumoniae* and *P. multocida* infections in swine respiratory diseases. Moreover, the prevalence of small plasmids with similar structure in various isolates of *Pasteurellaceae* bacteria from different regions revealed that this kind of plasmids are important cargos for the transmission of not only *floR* but also other resistance genes in porcine *Pasteurellaceae* pathogens. Continuous monitoring of florfenicol resistance and prevalence of *floR* gene and associated plasmids in *Pasteurellaceae* pathogens of veterinary origin are warranted, as this information is important for the control of infections caused by pathogenic species of *Pasteurellaceae* in food-producing animals.

## Data availability statement

The datasets presented in this study can be found in online repositories. The names of the repository/repositories and accession number(s) can be found in the article/supplementary material.

## Ethics statement

Ethical review and approval was not required for the animal study because the study was performed with resistant bacteria. Samples were taken by authorized veterinarians working in the farms. The researchers did not perform any animal studies.

## Author contributions

BL and LX designed and supervised the study. WZ, YQ, KL, JW, ZL, QS, and ZM provided resources for this study. XY and QS laboratory analysis and statistical analysis. BL and XY wrote the manuscript. All authors contributed to this article and approved the submitted version.
